# Alzheimer's Disease Classification Through Imaging Genetic Data With IGnet

**DOI:** 10.3389/fnins.2022.846638

**Published:** 2022-03-03

**Authors:** Jade Xiaoqing Wang, Yimei Li, Xintong Li, Zhao-Hua Lu

**Affiliations:** ^1^Department of Biostatistics, St. Jude Children's Research Hospital, Memphis, TN, United States; ^2^Department of Linguistics, The Ohio State University, Columbus, OH, United States

**Keywords:** Alzheimer's disease diagnosis, imaging genetics, deep learning, CNN, transformer, classification

## Abstract

The application of deep learning techniques to the detection and automated classification of Alzheimer's disease (AD) has recently gained considerable attention. The rapid progress in neuroimaging and sequencing techniques has enabled the generation of large-scale imaging genetic data for AD research. In this study, we developed a deep learning approach, IGnet, for automated AD classification using both magnetic resonance imaging (MRI) data and genetic sequencing data. The proposed approach integrates computer vision (CV) and natural language processing (NLP) techniques, with a deep three-dimensional convolutional network (3D CNN) being used to handle the three-dimensional MRI input and a Transformer encoder being used to manage the genetic sequence input. The proposed approach has been applied to the Alzheimer's Disease Neuroimaging Initiative (ADNI) data set. Using baseline MRI scans and selected single-nucleotide polymorphisms on chromosome 19, it achieved a classification accuracy of 83.78% and an area under the receiver operating characteristic curve (AUC-ROC) of 0.924 with the test set. The results demonstrate the great potential of using multi-disciplinary AI approaches to integrate imaging genetic data for the automated classification of AD.

## 1. Introduction

Alzheimer's disease (AD), the most common cause of dementia, is the sixth leading cause of death in the United States. It is an irreversible and progressive brain disorder that slowly destroys memory, thinking skills, eventually, and the ability to carry out the simplest tasks. AD is characterized by the loss of neurons and synapses in the cerebral cortex and in certain subcortical regions. A probable diagnosis can be made based on medical tests. However, initial AD symptoms are often mistaken for normal aging. A definite diagnosis usually requires an examination of brain tissue after death. Although no cure for AD has yet been found, treatments are available to slow its progress and improve the quality of life for patients. Therefore, accurate and timely diagnosis of AD is of great importance. Neuroimaging is among the most promising areas of research focused on early detection of AD, because microscopic changes in the brain begin long before the first signs of memory loss appear. In the present study, we focused on magnetic resonance imaging (MRI), a non-invasive structural imaging technique that can be performed comparatively easily and has been used extensively for clinical diagnosis and medical research. Structural imaging provides information on the shape, position, and volume of brain tissue. Although scientists have not yet reached a consensus, it has been shown that the brains of patients with AD shrink significantly as the disease progresses. Structural imaging research also has shown that shrinkage in specific brain regions, such as the hippocampus, may be an early sign of AD. The search for genetic risk factors for AD has made substantial progress over the years. For example, the ϵ_4_ allele of apolipoprotein E (APOE) has been identified as a strong genetic risk factor for both early-onset and late-onset AD (Farrer et al., 1997). In addition to the well-established effects of APOE, genome-wide association studies (GWASs) have identified more than 30 genomic loci that are associated with increased risk of AD (MacArthur et al., 2017). These advances in AD genetics have not only provided important assistance in AD diagnosis but have also encouraged current endeavors in translational research and personalized treatment of AD. Therefore, it is important and beneficial to facilitate AD diagnosis by leveraging both imaging and genetic data.

Our study was motivated by the Alzheimer's Disease Neuroimaging Initiative (ADNI). This began in 2004 and is the first “Big Data” project for AD. Comprehensive biomarkers, including T1 MRI and single-nucleotide polymorphism (SNP) data were collected for normal controls and for patients with AD. We aim to fuse imaging and genetic data by using deep learning techniques to build an automated AD classification system. This is a challenging task because of (a) the multi-modal nature of the data, in which brain MRI scans are three-dimensional (3D) images and genetic data are usually one-dimensional (1D) sequences; (b) the high dimensionality, whereby an MRI scan usually comprises hundreds of thousands of voxels and the genetic data can include millions of variants depending on the sequencing technique; and (c) the limited sample sizes, which are much smaller than the dimensions of the imaging genetic data.

Deep learning methods have been adopted for various biomedical applications, especially for handling complex high-dimensional data such as medical images (Kamnitsas et al., 2017), electronic health records (Lu et al., 2021), and omics data (Jumper et al., 2021). Convolutional neural networks (CNNs) have achieved great success with hierarchical feature representation in various challenging natural image recognition tasks, such as object detection (Li et al., 2015) and image classification (Krizhevsky et al., 2012). Lately, 3D CNNs have also demonstrated outstanding effectiveness in 3D medical image detection (Dou et al., 2016) and spatiotemporal feature learning (Tran et al., 2015). For 3D brain MRI, a 3D CNN, which uses 3D convolution kernels, has proved to be a more promising and reliable approach that takes full advantage of spatial contextual information in volumetric data for more accurate detection and prediction (Ueda et al., 2019). Recurrent neural networks, i.e., long short-term memory neural networks (Hochreiter and Schmidhuber, 1997) and gated recurrent neural networks (Chung et al., 2014), have been established as effective approaches for sequence modeling. They have also been applied to omics studies, such as the detection of DNA base modifications (Liu et al., 2019) and protein–RNA binding prediction (Li et al., 2019). Lately, Transformer (Vaswani et al., 2017), a model architecture that eschews recurrence and instead relies entirely on an attention mechanism, has been widely adopted in various fields. Transformer was originally proposed as a sequence-to-sequence model for machine translation (Sutskever et al., 2014). Subsequent work has shown that Transformer-based pre-trained models can achieve state-of-the-art performance in various tasks (Qiu et al., 2020). Transformer has also been adopted in omics-related tasks such as protein sequence modeling (Rives et al., 2021) and DNA sequence modeling (Ji et al., 2021).

Recently, there has been a dramatic proliferation of multi-disciplinary artificial intelligence (AI) tasks that combine AI techniques in more than one domain, such as tasks that require an inter-section of vision and language (Fang et al., 2015; Vinyals et al., 2015). One example is visual question answering (VQA) (Antol et al., 2015). Given an image and an open-ended, natural language question about the image, the task is to provide an accurate natural language answer. This requires fusing computer vision (CV) techniques (for the input image) and natural language processing (NLP) techniques (for the input question) to generate the output (the answer sentence) jointly. Motivated by the VQA system, we are interested in combining brain MRI and genetic variant information to predict AD diagnosis by using deep neural network models. The brain MRI scans are treated as 3D images, and the genetic variants, i.e., the SNPs, are treated as 1D sequences. Our goal is to make full use of the imaging genetic data to achieve automated AD classification.

In this article, we present an imaging genetic deep neural network system (IGnet) that fuses imaging and genetic inputs to generate a binary AD diagnosis. The proposed method is based on the following components: an imaging channel using a 3D CNN for the 3D imaging input; a genetic channel using a Transformer encoder for the SNP sequence input; and a multi-layer perceptron (MLP) for fusing the two channels and for generating the AD classification label. We have applied the proposed approach to the ADNI data set for automated detection of patients with AD vs. normal controls, using baseline T1 brain MRI and SNPs on chromosome 19. The main contributions of this article are as follows: (a) we present IGnet, which combines CV and NLP deep learning techniques to use 3D brain image data and genetic sequence data jointly to predict AD diagnosis; (b) we show that fusing brain MRI and genetic information together can provide predictions that are more accurate than those obtained with a single data modality; and (c) we show that the proposed method can serve as a baseline for related imaging genetic tasks and that it can be easily generalized.

## 2. Materials and Methods

### 2.1. ADNI Data

The development of IGnet was motivated by the imaging genetic data analysis of the ADNI data set. ADNI initially recruited approximately 800 participants (ADNI-1) according to its initial aims and was extended by three follow-up studies, namely, ADNI-GO, ADNI-2, and ADNI-3. Our experiments were carried out on an ADNI-1 data subset with 379 participants who are either AD patients or cognitive normal controls (MCI participants were excluded) and with both baseline 1.5T MRI and genetic information available. We aim to achieve automated AD classification by using baseline T1 MRI and genetic variant data from the 379 ADNI-1 participants, comprising 174 patients with AD and 205 normal controls. The MRI data, which were collected across various 1.5-Tesla MRI scanners with protocols individualized for each scanner, included standard T1-weighted images obtained using volumetric 3D sagittal MPRAGE or equivalent protocols with varying resolution. The typical protocol included the following variables: repetition time (TR) = 2,400 ms, inversion time (TI) = 1,000 ms, flip angle = 8^*o*^, and field of view (FOV) = 24 cm, with a 256 × 256 × 170 acquisition matrix in the x-, y-, and z- dimensions, yielding a voxel size of 1.25 × 1.26 × 1.2 mm (Jack et al., 2008; Bühlmann et al., 2013). The MRI data were preprocessed by standard steps, including anterior commissure and posterior commissure correction, skull stripping, cerebellum removal, intensity inhomogeneity correction, and registration, and were finally down-sampled to 128 × 128 × 128. For the genetic data, the Human 610-Quad BeadChip (Illumina, Inc., San Diego, CA) was used to genotype the ADNI participants, which resulted in a set of 620,901 SNP and copy number variation (CNV) markers. Because the APOE SNPs, rs429358 and rs7412, are not included on the Human 610-Quad Bead-Chip, they were genotyped separately. These two SNPs together define a three-allele haplotype, namely the ϵ_2_, ϵ_3_, and ϵ_4_ variants, and the ADNI database recorded whether these variants were present in a given individual. The software EIGENSRAIT in the package of EIGENSOFT 3.0 was used to calculate the population stratification coefficients of all subjects.

### 2.2. Method

**Figure 1:**
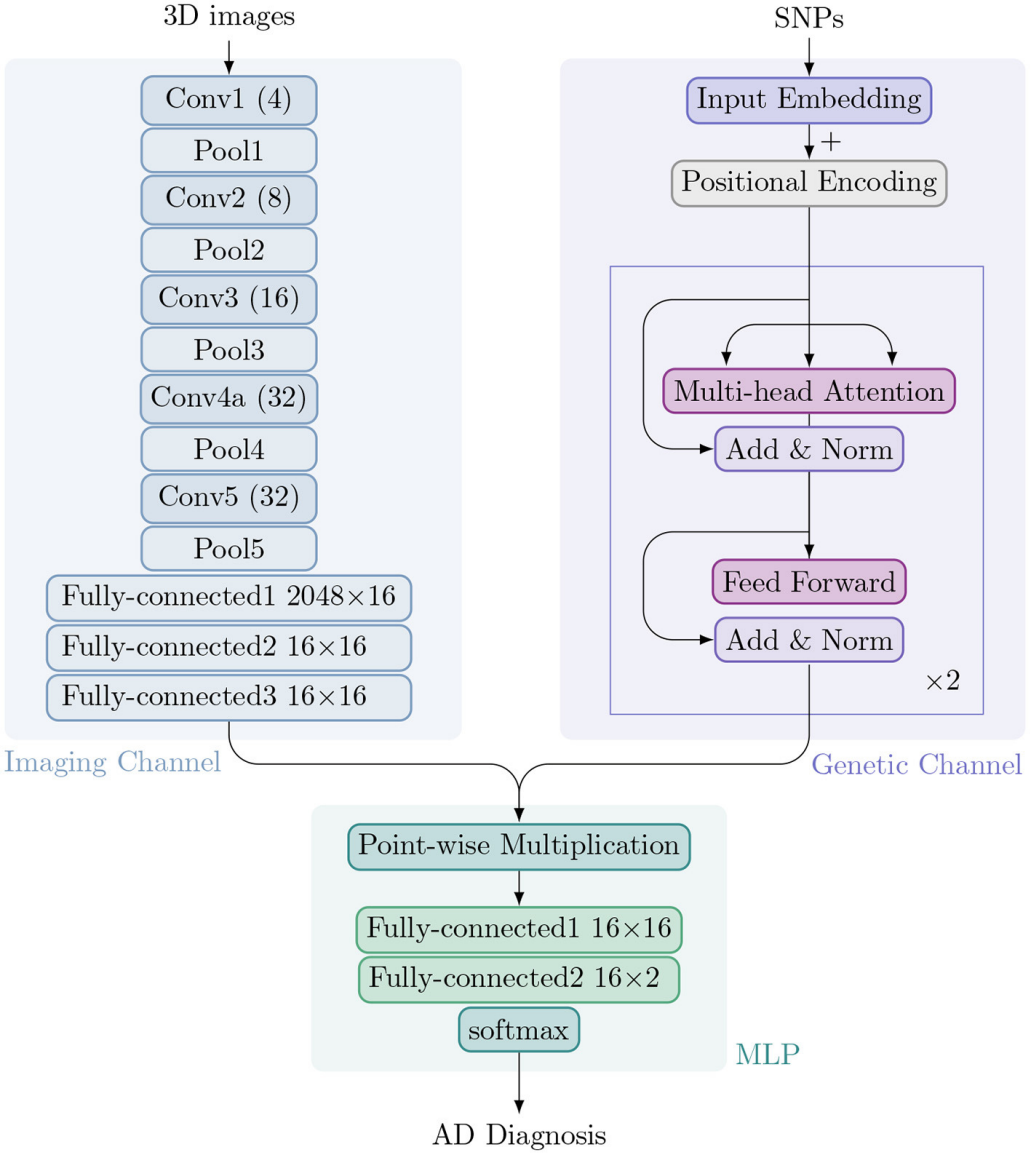
Architecture of IGnet. Upper left: The imaging channel with a 3D CNN. Upper right: The genetic channel with a Transformer encoder. Bottom: The MLP with two fully connected layers followed by softmax.

We have developed IGnet, a two-channel 3D imaging genetic network culminating with a softmax over *K* possible outputs. In the ADNI, *K* = 2 for AD diagnosis (normal control or patient with AD). Figure 1 shows an overview of the proposed approach, which is composed of two channels: an imaging channel with a 3D CNN and a genetic channel with a Transformer encoder. After acquiring the imaging embedding and genetic embedding in each channel separately, the two embeddings are combined and the combined embedding is then passed to an MLP for the final classification. Figure 1 shows the components of IGnet.

#### 2.2.1. Imaging Channel

Typically, a (2D) CNN (Goodfellow et al., 2016) alternately stacks convolutional and sub-sampling, e.g., max-pooling, layers. In a convolutional layer, small feature extractors (kernels) sweep over the topology and transform the input into feature maps. In a max-pooling layer, activations within a neighborhood are abstracted to acquire invariance to local translations. After several convolutional and max-pooling layers, feature maps are flattened into a feature vector (imaging embedding). For classification tasks using image data alone, the feature vector is usually passed to fully connected layers, after which a softmax classification layer yields the prediction probability.

Compared to a 2D CNN, a 3D CNN can model 3D images better owing to its 3D convolution and 3D pooling operations (Zou et al., 2017). In our proposed IGnet, the network for the imaging channel is a 3D CNN that is set up to take preprocessed 3D brain MRI as input. With limited GPU memory and sample size, we designed the 3D CNN to have five convolution layers and five pooling layers (each convolution layer is immediately followed by a pooling layer). The numbers of filters for the five convolution layers are [4, 8, 16, 32, 32] for layers 1 to 5, respectively, as shown in Figure 1. As suggested by Tran et al. (2015); Dou et al. (2016), all of the 3D convolution filters are 3 × 3 × 3 with stride 1 × 1 × 1. All of the 3D pooling layers are max pooling, with kernel size 2 × 2 × 2 with stride 1 × 1 × 1. The imaging channel concludes with three fully connected layers that yield the final imaging embedding of dimension 16.

#### 2.2.2. Genetic Channel

Attention mechanisms have become an integral part of compelling sequence modeling and transduction models in various tasks, enabling the modeling of dependencies without regard to their distance in the input or output sequences (Bahdanau et al., 2014; Kim et al., 2017). Transformer is a model architecture that relies entirely on an attention mechanism to draw dependencies between input and output (Vaswani et al., 2017). It also allows for significant parallelization and has been shown to be computationally efficient in many tasks, such as translation.

In the proposed method, we use a two-layer Transformer encoder in which each layer consists of multi-head self-attention (with four heads), residual connection, layer normalization, and a fully connected layer. The input genetic sequence is processed to a genetic embedding of dimension 16, which is the same dimension as for the imaging embedding.

#### 2.2.3. Multi-Layer Perceptron (MLP)

The image and genetic embeddings are combined to obtain a single embedding *via* element-wise multiplication. This combined embedding is then passed to an MLP, a fully connected neural network classifier with two hidden layers and 16 hidden units in each layer with ReLU non-linearity, followed by a softmax layer to obtain a distribution over a binary distribution for normal controls or patients with AD.

### 2.3. Implementation

**Figure 2:**
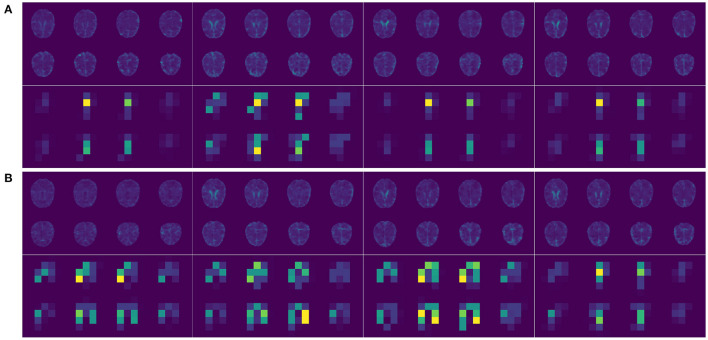
**(A)** Upper panel, selected 2D slices of the input 3D brain images of 4 randomly picked AD patients; lower panel, the corresponding feature maps after 5 convolution layers (two selected filters). **(B)** Upper panel, selected 2D slices of the input 3D brain images of 4 randomly picked normal controls; lower panel, the corresponding feature maps after 5 convolution layers (two selected filters).

**Figure 3:**
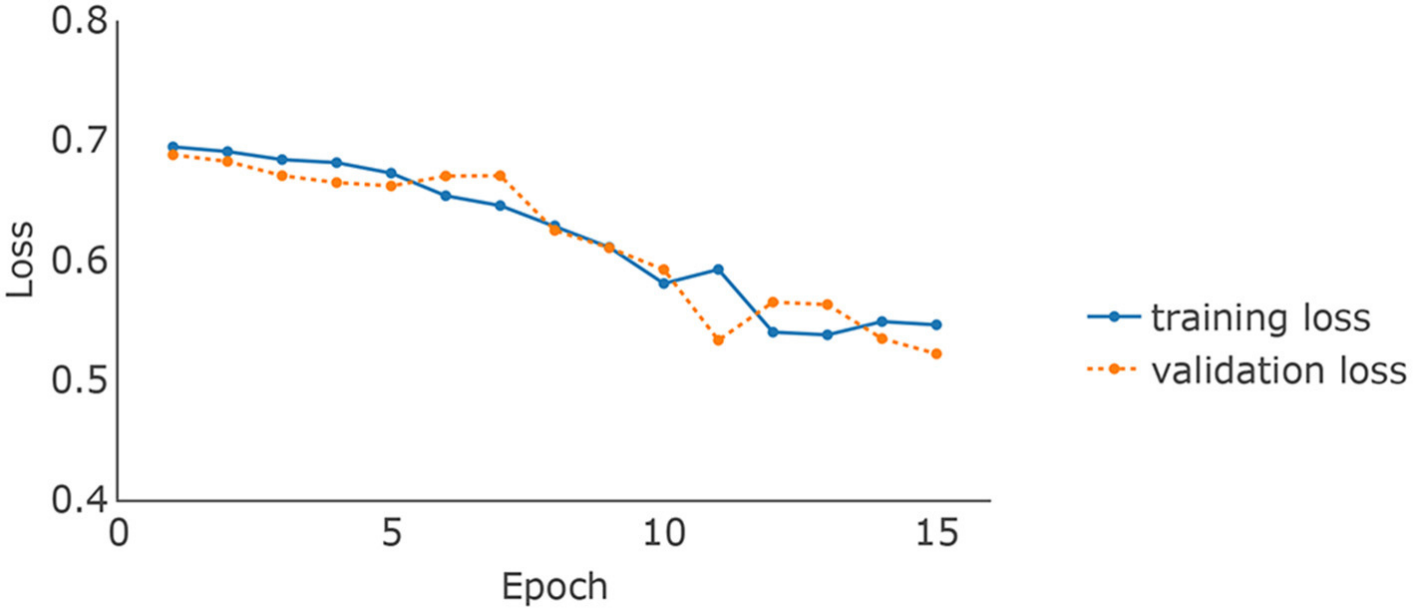
The changes of training and validation losses overtime of IGnet.

The brain MRI scans from the ADNI were preprocessed to dimensions 128 × 128 × 128, where the value at each voxel was an integer indicating a grayscale intensity from 0 to 255. Examples of the preprocessed brain images of 4 randomly picked AD patients and normal controls are shown in Figures 2A,B upper panel, respectively. We focused on the 8946 SNPs on chromosome 19. For each SNP, we conducted Fisher's exact test with the binary AD diagnosis. Ninety-eight SNPs with P values < 0.01 were selected and concatenated with APOE ϵ_4_, where the value for each SNP was {0,1,2}. Because APOE ϵ_4_ was genotyped separately in the ADNI, we used a different set of input embeddings for the number of alleles of APOE ϵ_4_ from all of the other 98 selected SNPs on chromosome 19. The data set was randomly split into training, validation, and testing sets, with 80%, 10%, and 10% samples, respectively. The ratios of the number of AD patients vs. normal controls on the three subsets are around 0.85. We used the categorical cross-entropy loss with *K* = 2 categories. The loss function was optimized by AdamW (Loshchilov and Hutter, 2017), where β_1_ = 0.9, β_2_=0.999, and ϵ = 10^−8^. The batch size was 16. The initial learning rate was 0.001. For the hyperparameters, the number of layers of the imaging channel was chosen based on (Tran et al., 2015) except that we only kept one of every two consecutive convolution layers. We applied dropout (Srivastava et al., 2014) to the output of each sub-layer. The dropout rate of the imaging channel was set to be 0.2 as suggested by Dou et al. (2016). Examples of the feature maps after 5 convolution layers of the 4 AD patients and normal controls are shown in Figures 2A,B lower panel. One widely used dropout rate for Transformer encoders is 0.1. However, Araabi and Monz (2020) suggests using 0.3 under low-resource conditions. Given the limited sample size of our data set, we choose to use a dropout rate 0.2 between 0.1 and 0.3 for the genetic channel. The other tuning parameters such as the number of nodes in the fully connected layers and the number of filters in the 3D CNN were optimized based on the training and validation sets. For the computation resources, the imaging and the genetic data together take up around 256M for each batch and the model parameter size is 0.58M. Therefore training the IGnet requires less than 300M of memory which should be compatible with commonly used GPUs. We conducted our experiments with a Tesla V100. The training of IGnet took around 45min where the optimization stopped at approximately 13 epochs with patience 3. The changes of the training and validation losses over time are shown in Figure 3.

## 3. Results

We evaluated the performance of IGnet on the test set by calculating the accuracy (the number of participants correctly classified as patients with AD or normal controls divided by the total number of participants), the precision (the number of patients correctly classified as having AD divided by the total number of patients classified as having AD), the recall (the number of patients correctly classified as having AD divided by the number of patients that actually had AD), the F1 score (equal to the harmonic mean of precision and recall), the AUC-ROC (the area under the receiver operating characteristic curve), and the AUC-PRC (the area under the precision recall curve). We compared IGnet to two restricted versions that used genetic data alone (IGnet-G) or imaging data alone (IGnet-I). For IGnet-G, only the genetic embedding was passed to the MLP and the image channel was excluded. IGnet-I worked in a similar manner; only the imaging embedding was passed to the MLP and the genetic channel was excluded. All other implementation details for IGnet-G and IGnet-I, such as the hyperparameters, batch size, and dropout rates, were the same as IGnet, as described in section Implementation.

We evaluated the performances of the two important building blocks namely the imaging channel and the genetic channel by comparing IGnet-I and IGnet-G to several popular single modality based prediction methods. IGnet-I is essentially a 3D CNN which has been shown to be effective on handling 3D volumetric medical data. We compared IGnet-I to two conventional methods including support-vector machines (SVMs) and functional principal component analysis (FPCA). For the SVMs, we adopted the pipeline proposed by Varatharajah et al. (2019). It first conducts feature selection using joint mutual information (JMI) and then the selected top k features are passed to an SVM classifier. We set *k* = 100 and considered both linear (SVM-linear) and radial (SVM-radial) kernels where the hyperparameters were chosen based on 10-fold cross validation. For the FPCA based method, we considered the idea proposed by Wang et al. (2021). We first used FPCA for feature extraction and then applied logistic Lasso regression to the top 10 PCs to build the prediction model. IGnet-G is essentially a Transformer encoder that has shown to be effective on many task especially on sequential data. We compared IGnet-G to several popular methods including recurrent neural network (RNN, Liu et al., 2016), ridge regression (Reg-ridge), and Lasso regression (Reg-lasso). More specifically, the RNN that we used was a single layer gated recurrent unit network (GRU, Cho et al., 2014) where the hidden size was set to be the same as IGnet-G. The tuning parameters in the ridge regression and Lasso regression models are selected based on 10-fold cross validation. In order to have a better understanding of how much the APOE ϵ_4_ and other SNPs in chromosome 19 contribute to the AD classification, we evaluated the performance of the aforementioned 4 methods without including APOE ϵ_4_. We also evaluate the performance of the MLP by comparing to two simple ensembling of IGnet-G and IGent-I methods, one by simply averaging the prediction probabilities of IGnet-G and IGnet-I (IG-avg), another by majority vote (IG-vote) where participants are classified as AD patient only if they are classified as AD by both IGnet-G and IGnet-I.

**Figure 4:**
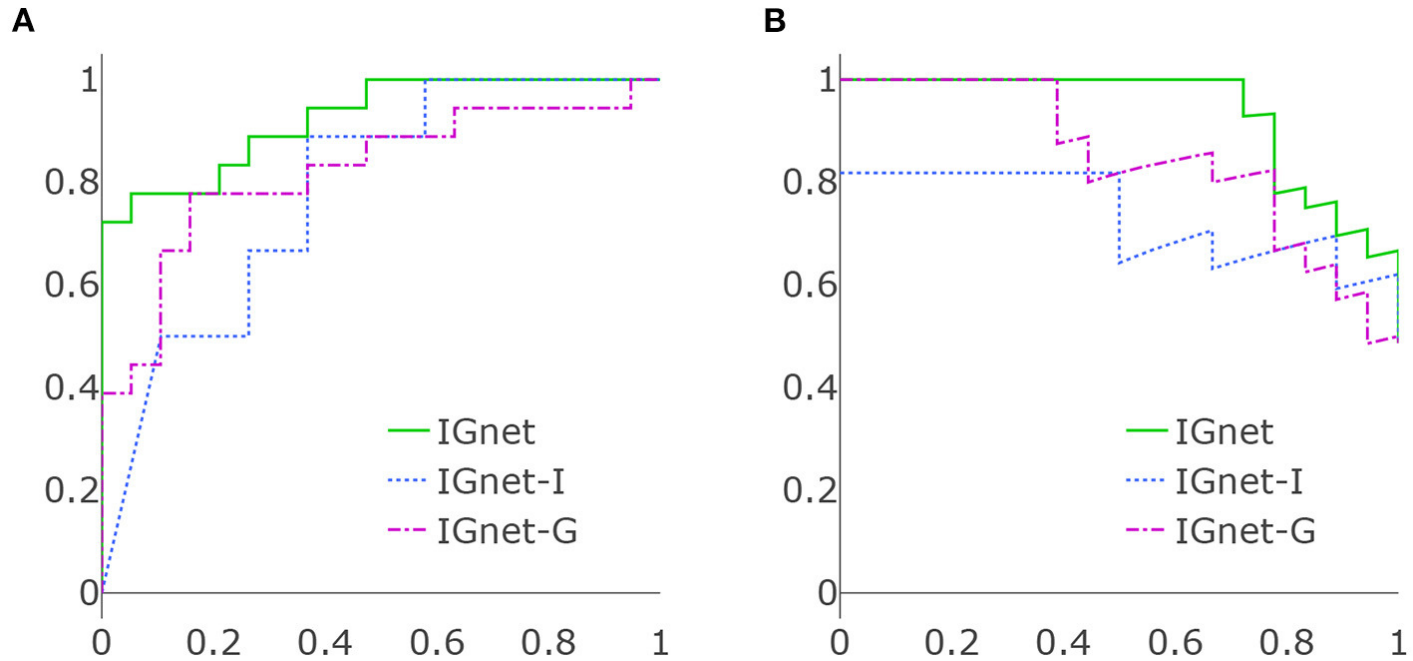
**(A)** ROC curves of IGnet, using both imaging and genetic inputs; IGnet-I, using imaging input alone; and IGnet-G, using genetic input alone. **(B)** PRC curves of IGnet, IGnet-I, and IGnet-G.

Table 1 presents the results of applying all the aforementioned methods to the ADNI data set. For the methods using both imaging and genetic data, IGnet yields the highest AD detection accuracy of 83.78%, indicating the superiority of the proposed two channel-approach on AD classification. For the methods using imaging data only, as expected, IGnet-I achieves the highest accuracy of 67.57% that coincides with the literature suggesting 3D CNN being more suitable for 3D volumetric data. For the methods using genetic data, IGnet-G gives the highest accuracy of 78.38% followed by RNN with 64.86%. When excluding the APOE ϵ_4_, the accuracy of all the four methods were dropped. IGnet-G and RNN performed comparatively well where the RNN is slightly better than IGnet-G. The ROC and PRC curves for IGnet, IGnet-G, and IGnet-I are shown in Figure 4. Both Figure 4 and the AUC-ROC and AUC-PRC values in Table 1 show that IGnet outperforms IGnet-G and IGnet-I, further indicating that integrating imaging and genetic data can boost the accuracy of AD classification.

**Table 1:** Comparison of the performance of IGnet on the ADNI data set. AUCs are not available for the IG-vote, SVM-linear, and SVM-radial methods, therefore, are not presented.

	**Accuracy**	**Precision**	**Recall**	**F1 score**	**AUC-ROC**	**AUC-PRC**
***Methods using both imaging and genetic data:***						
IGnet	83.78%	87.50%	77.78%	0.824	0.924	0.935
IG-avg	81.08%	82.35%	77.78%	0.800	0.886	0.893
IG-vote	70.27%	88.89%	44.44%	0.593	–	–
***Methods using imaging data:***						
IGnet-I	67.57%	68.75%	61.11%	0.647	0.784	0.737
SVM-linear	64.86%	50.00%	38.46%	0.435	–	–
SVM-radial	62.16%	46.67%	53.85%	0.500	–	–
FPCA	62.16%	52.94%	60.00%	0.563	0.676	0.655
***Methods using genetic data:***						
IGnet-G	78.38%	77.78%	77.78%	0.778	0.822	0.845
RNN	72.97%	76.92%	55.56%	0.645	0.839	0.850
Reg-ridge	70.27%	62.50%	66.67%	0.645	0.748	0.640
Reg-lasso	67.57%	52.63%	76.92%	0.625	0.744	0.609
***Methods using genetic data without APOE*** **ϵ_4_:**						
IGnet-G	67.57%	71.43%	55.56%	0.625	0.827	0.823
RNN	70.27%	57.14%	61.54%	0.593	0.837	0.749
Reg-ridge	62.16%	52.94%	60.00%	0.563	0.733	0.634
Reg-lasso	64.86%	50.00%	84.62%	0.629	0.750	0.668

## 4. Discussion

We have developed a deep learning approach, IGnet, that combines CV and NLP techniques. It enables 3D image and 1D sequence inputs to be combined for automated classification. The method was applied to the ADNI data set, with both imaging and genetic data being used to classify patients with AD vs. normal controls. The accuracy of IGnet is superior to that obtained with a single input modality, highlighting the superiority of integrating imaging and genetic information for AD classification.

The proposed method has many potential extensions. Besides 3D CNN, 3D residual networks (ResNet, He et al., 2016) have also performed strongly in image-related tasks such as video action recognition (Feichtenhofer and P., 2016) and brain segmentation (Chen et al., 2018). It is worth investigating whether 3D ResNet can further improve the classification performance. The proposed method was applied to the preprocessed genotyping data of the ADNI database, in which each SNP was coded with {0,1,2}. Lately, Transformer has proved to be effective at handling raw sequencing data, such as DNA sequences coded with ATCG (Ji et al., 2021). However, distinct from other sequences such as natural language, DNA sequences are long and shallow. The sequence length can easily exceed tens of thousands, whereas the bag of words always contains only four distinct elements {A,T,C,G}. This could potentially be one of the reasons why the IGnet-G performs inferior to RNN when excluding APOE ϵ_4_ on our ADNI data set. Therefore, techniques such as sparse attention (Zaheer et al., 2020) that alleviate the memory and computational cost and K-mer representation of sequences (Nahum et al., 2021) should be considered. It would be useful to investigate whether using the ATCG data with a properly designed Transformer encoder can boost the classification accuracy for AD. IGnet was trained from scratch using around 300 samples. The model capacity, such as the hidden sizes, was chosen to be smaller than that of widely applied CNNs and transformers. As with other tasks with limited training data, pre-training on a similar dataset might improve the performance and is, therefore, also worth further investigating.

## Data Availability Statement

Publicly available datasets were analyzed in this study. This data can be found at: http://adni.loni.usc.edu/.

## Author Contributions

JW and XL performed data analysis. All authors contributed to the conception and design of the study and participated in drafting and editing the manuscript.

## Funding

This research work of YL, Z-HL, and JW was in part supported by the Department of Biostatistics at St. Jude Children's Research Hospital (SJCRH) and the American Lebanese Syrian Associated Charities (ALSAC). JW's research was also partly supported by the Academic Office Program at SJCRH.

## Conflict of Interest

The authors declare that the research was conducted in the absence of any commercial or financial relationships that could be construed as a potential conflict of interest.

## Publisher's Note

All claims expressed in this article are solely those of the authors and do not necessarily represent those of their affiliated organizations, or those of the publisher, the editors and the reviewers. Any product that may be evaluated in this article, or claim that may be made by its manufacturer, is not guaranteed or endorsed by the publisher.
